# Are the unken reflex and the aposematic colouration of Red-Bellied Toads efficient against bird predation?

**DOI:** 10.1371/journal.pone.0193551

**Published:** 2018-03-29

**Authors:** Debora Wolff Bordignon, Valentina Zaffaroni Caorsi, Patrick Colombo, Michelle Abadie, Ismael Verrastro Brack, Bibiana Terra Dasoler, Márcio Borges-Martins

**Affiliations:** 1 Programa de Pós-Graduação em Biologia Animal, Departamento de Zoologia, Instituto de Biociências, Universidade Federal do Rio Grande do Sul, Porto Alegre, Rio Grande do Sul, Brasil; 2 Museu de Ciências Naturais, Fundação Zoobotânica do Rio Grande do Sul, Porto Alegre, Rio Grande do Sul, Brasil; 3 Programa de Pós-Graduação em Ecologia, Instituto de Biociências, Universidade Federal do Rio Grande do Sul, Porto Alegre, Rio Grande do Sul, Brasil; University of Sussex, UNITED KINGDOM

## Abstract

Aposematic signals as well as body behaviours may be important anti-predator defences. Species of the genus *Melanophryniscus* are characterised by having toxic lipophilic alkaloids in the skin and for presenting a red ventral colouration, which can be observed when they perform the behaviour called the unken reflex. Both the reflex behaviour and the colouration pattern are described as defence mechanisms. However, there are currently no studies testing their effectiveness against predators. This study aimed to test experimentally if both ventral conspicuous colouration and the unken reflex in *Melanophryniscus cambaraensis* function as aposematic signals against visually oriented predators (birds). We simulated the species studied using three different clay toad models as follows: (a) in a normal position with green coloured bodies, (b) in the unken reflex position with green coloured body and extremities and (c) in the unken reflex position with a green body and red extremities. Models were distributed on a known *M*. *cambaraensis* breeding site and in the adjacent forest. More than half of the attacks on the models were from birds; however, there was no preference for any model type. Thus, just the presence of the red colour associated with the motionless unken reflex position does not seem to prevent attacks from potential predators. It is possible that the effective aposematic signal in *Melanophryniscus* is achieved through the unken reflex movement together with the subsequent exhibition of the warning colouration and the secretion of toxins.

## Introduction

Antipredator strategies encompass several mechanisms involving the behaviour, morphology and colouration of prey species, which evolve either to avoid detection (e.g. camouflage) or to enhance honest or dishonest unprofitability signaling (e.g. aposematism, masquerade, pursuit deterrence, deflection, and deimatism) [1,2]. Organisms that contain toxic chemical substances may exhibit conspicuous colourations as visual honest signals of their toxicity [3,4]. Aposematic colouration is a common trait in nature which serves to warn potential predators that an individual is unpalatable, harmful, or potentially dangerous and should be avoided [5–8]. The effectiveness of aposematic signals depends on the ability of predators to associate the conspicuous colouration of a prey species with the disadvantage of attacking that species [8–10].

Aposematic colouration can be associated with behavioural signals to enhance its effect. Several prey postures, movements or sounds can cause fear responses in predators, the so-called deimatism [2]. These displays could cause predators to misclassify a potential threat by giving the impression of a larger body size or by displaying body areas which contain higher concentrations of toxic substances, for example [1,2,11]. A behaviour shared by different amphibian families is the unken reflex which was first described for *Bombina bombina* [12]. In the unken reflex, the individual arches its body, raising the head and the posterior region to reveal hidden areas with aposematic colouration [12,13]. During these behaviors individuals usually close their eyes or cover them with their hands [13,14], the extremities of the body are exposed, and the ventral area of the hands, feet and throat are displayed. The unken reflex posture is also generally associated with aposematic ventral colourations and with the presence of toxic substances [15]. This behaviour has been reported in many amphibian species [16,17], including the anuran genera: *Bombina* (Bombinatoridae) [18], *Melanophryniscus* (Bufonidae) [18–21], *Hemisus* (Hemisotidae) [22], *Boana* and *Smilisca* (Hylidae) [23,24], *Neobatrachus* (Limnodynastidae) [25], *Boophis* (Mantellidae) [26], *Pseudophryne* (Myobatrachidae) [25], *Rana* (Ranidae) [27–31] and *Nyctixalus* and *Rhacophorus* (Rhaphocoridae) [32–34]. In urodeles, the unken reflex occurs in some Salamandridae such as *Lissotriton* [35], *Salamandrina* [36,37], *Taricha* [38] and *Triturus* [39]. However, behaviour terminology is evidently not uniform and many of the reported species lack some behavioural or morphological attributes of the typical unken reflex as seen in *Bombina* and *Melanophryniscus*. The unken reflex seems to have been confounded with other behaviours (e.g. eye-protection), particularly in species lacking aposematic ventral colouration or toxic substances [17].

Due to the display of aposematic colouration, it is generally assumed that the unken reflex may be efficient primarily against visually oriented predators. It is known that birds possess one of the most elaborate mechanisms of colour vision within the vertebrates [40,41] and may represent the main predators of several aposematic species, including the poison frogs of the Dendrobatidae family. However, predation events are very difficult to observe in nature and there is still little experimental evidence of predator attacks on this family [42]. A convenient experimental method used in short-term studies of predation consists of recording attack marks on soft replicas (such as clay models) of the species of interest [43,44]. Yet despite this, there are currently no studies of predation on anuran species which display ventral aposematic colouration during the unken reflex.

Using clay models of the south American red-bellied-toad *Melanophryniscus* c*ambaraensis* the present study aimed to experimentally test whether conspicuous colouration and the unken reflex function as effective aposematic signals against visually oriented predators (i.e. birds). If both strategies are effective and independent defence mechanisms, we expect to find different rates of predation attempts related to the interaction between these strategies.

## Materials and methods

### Species model

The Bufonidae genus *Melanophryniscus* Gallardo, 1961 includes 29 species [45] restricted to southeastern South America [46], which are known as south American red-bellied toads. These small toads secrete skin toxins, such as alkaloids and bufadienolides (e.g. [47,48]) and when disturbed they display the unken reflex in which they expose their brightly coloured ventral areas [16,18,21,49,50]. The conspicuous ventral colouration and the unken reflex of this species have been suggested as probable defence mechanisms to avoid predation. However, their effectiveness has not yet been investigated. Previous studies describe only the behaviour itself, usually in response to human manipulation, and not in response to natural predation attempts (e.g. [16,21]).

**Fig 1:**
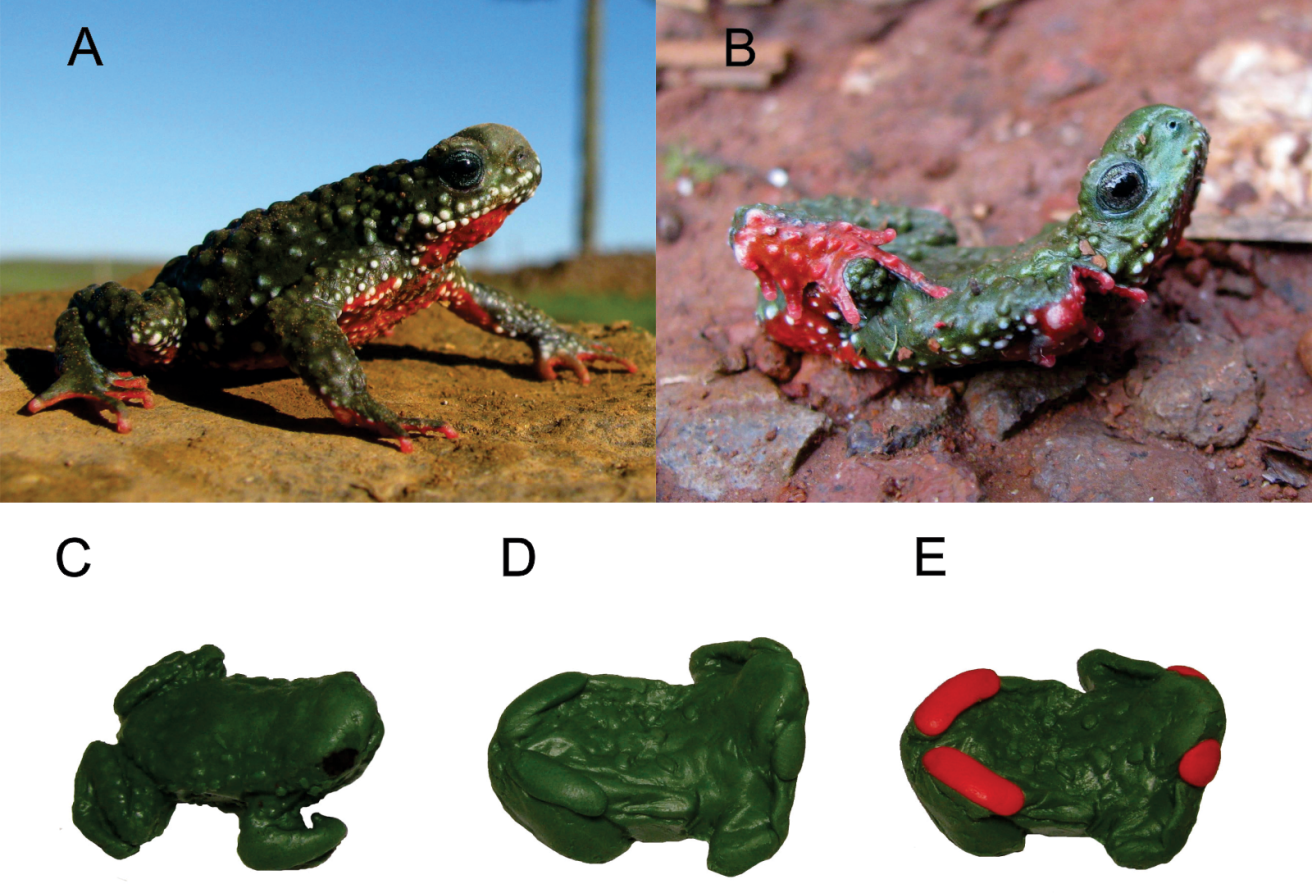
***Melanophryniscus cambaraensis*** (A) in normal position and (B) in unken reflex position displaying the aposematic colouration of its hands and feet (animal with open eyes, unlike previous descriptions). Clay model replicas of the species (C) in normal position, (D) with green hands and feet in unken reflex position and (E) with red hands and feet in unken reflex position.

*Melanophryniscus* c*ambaraensis* Braun & Braun, 1979 (Fig 1A and 1B) is one of the three species of the genus *Melanophryniscus* with green dorsal colouration, and with ventral colouration which may vary from red to orange [51–53]. This red-bellied-toad is a threatened, vulnerable species [54,55], endemic of the southern of the Mixed Ombrophilous Forest and of the northern of South Brazilian Grasslands of Rio Grande do Sul, Brazil [56]. The species is historically known only from two populations, located respectively in São Francisco de Paula and Cambará do Sul municipalities [57]. However, in the last locality it has not been seen since 1990 (according to several field expeditions conducted on this locality to search this toad). As with other species of the genus, *M*. *cambaraensis* exhibits explosive reproduction, without a defined reproductive season, breeding during a few days immediately after copious rains [56].

### Study area

We performed this study in the Floresta Nacional de São Francisco de Paula (FLONA SFP) (29° 25’ 41.3” S, 50° 23’ 44.5” W, 923 m above sea level). The FLONA SFP is a protected area of 1607 ha, covered mostly by the native Mixed Ombrophilous Forest [58]. The climate is Temperate Superhumid, with temperatures ranging from -3–18°C in the winter and 18.3–27°C in the summer, with average annual temperature of 14.5°C [59] and annual precipitation exceeding 2000 mm [60].

We conducted the experiment in May 2016 around the breeding site of the species. This site is located at the edge of a small, unpaved road, and is a rocky outcrop partially covered by a thin layer of vegetation (approximately 20 m long and 4 m wide). A planted forest of Parana pine, *Araucaria angustifolia*, composes the surrounding area. Toads have been recorded in this adjacent forest up to 120 m from the breeding site [56].

### Clay models

We made 900 models by hand which were phenotypically simillar to *Melanophryniscus cambaraensis* using pre-coloured, non-toxic plasticine modeling clay (Sculpey III® and Acrilex®). This clay is well suited for this type of study as it does not harden and retains markings left by predators [61]. We created two rubber moulds in two different positions: normal position (as animals are usually found in nature) and unken reflex position. These were created using two fixed specimens deposited in the herpetological collection of the Museu de Ciências Naturais of Fundação Zoobotânica of Rio Grande do Sul (MCN 13459 and unvouchered specimen), with snout-to-vent-lengths representative of the average size of the FLONA SFP population (SVL 32 ± 1,6; [62]). The clay models were organised into three categories: (a) green body and extremities in a normal position (normal) (Fig 1C), (b) green body and extremities in unken reflex position (green unken) (Fig 1D), and green body with red extremities in unken reflex position (red unken) (Fig 1E). For each category, we constructed 300 clay models, totalling 900 toads. A black permanent pen (Sharpie®) was used to draw the eyes only on the normal models, since animals in the unken reflex position usually close their eyes or cover them with their hands [13,14].

We collected one individual of *Melanophryniscus cambaraensis* in order to measure the reflectance of different colours and ultraviolet light, since birds have tetrachromatic vision and can perceive ultraviolet wavelengths [40,63]. These measurements were made using a spectrometer (“Ocean Optics”, version 2200 SD). We measured the wavelengths of light reflected from living and clay toads and compared the results. We mixed the clay to match the wavelengths reflected from the living animal as closely as possible (green: 550 nm; red: 650 nm) radiation intensity (green: 120 counts; red: 160 counts). Neither the animal nor the clay models reflected ultraviolet. The voucher specimen collected was deposited in the Coleção Herpetológica do Departamento de Zoologia from the Universidade Federal do Rio Grande do Sul (UFRGS.7317).

### Ethical and legal procedures

This study was authorised by the Instituto Chico Mendes de Conservação da Biodiversidade–ICMBio, under SISBIO (Sistema de Autorização e Informação em Biodiversidade) license number 52119–1.

### Experimental design

**Fig 2:**
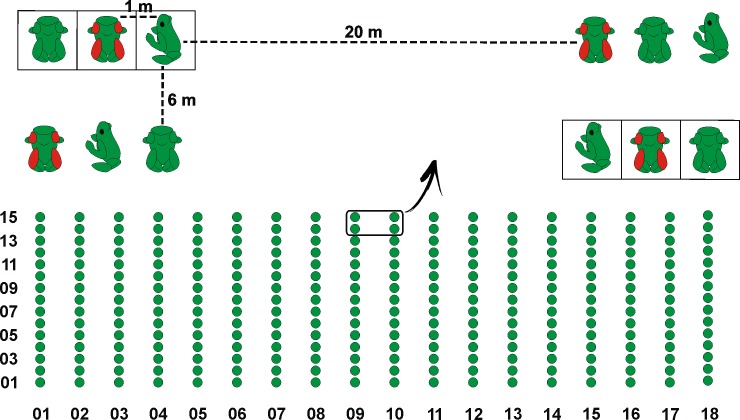
Experimental design. The 18 transects were divided into 15 blocks distributed every 6 m, containing the three model categories in parallel. Categories were randomly ordered and placed every 1 m inside the blocks. Half of the blocks were placed on the leaf litter and half onto a white plastic board.

A total of 810 out of 900 models were placed sequentially (temporally and spatially) into the forest. The experimental design was composed of 18 transects positioned perpendicular to the unpaved road. The middle transects were located next to the breeding site. Each transect was 90 m long and was separated by 20 m from other transects. Each transect had 15 blocks distributed every 6 m, containing the three model categories placed parallel to each other. Categories were randomly ordered and placed every 1 m inside the blocks (Fig 2). To remove possible cryptic effects [44], half of the blocks were placed on the leaf litter and half onto a white opaque plastic board (9 cm x 9 cm). We alternated each background between the blocks. On the same day, the remaining 90 models (30 of each category) were randomly distributed across the breeding site of the species and half of them selected at random were placed on a white background. In total the experiment lasted five days with the following design: Day 1 (models were placed) > Days 2–4 (models were exposed to the environment) > Day 5 (models were removed). During the environmental exposure of the models (72 hours) the area was not disturbed by anthropic activity. On the last day, the 900 models were carefully collected, photographed and classified with the type and number of attacks recorded.

### Data analyses

**Fig 3:**
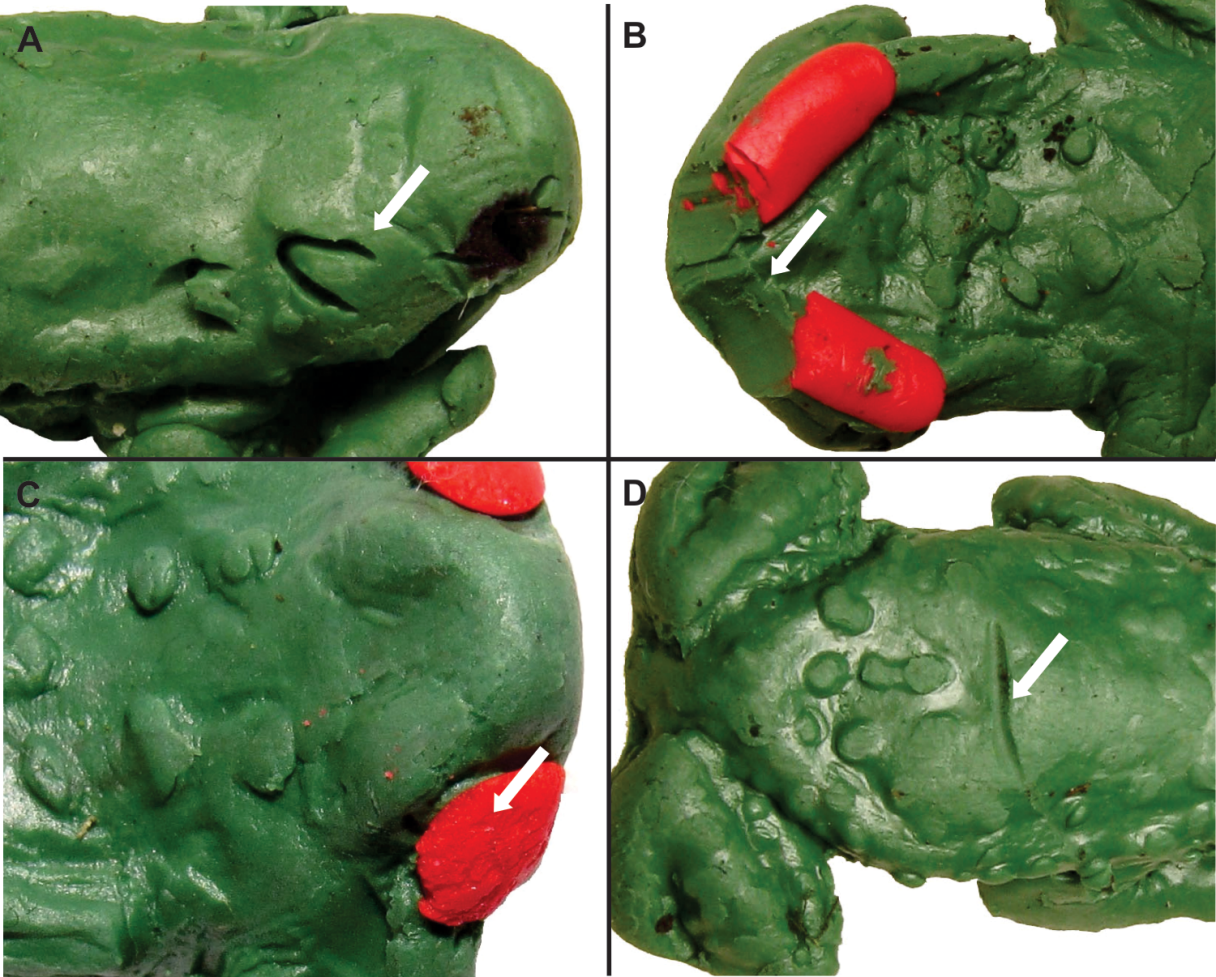
Examples of clay models attacked. (A) bird, (B) mammal, (C) arthropod, and (D) unidentified origin.

Initially, we classified models as attacked (when there were bite marks) or not attacked. Additionally, we recorded the position of the marks on the clay toads as either anterior, posterior or both. Consecutive attacks on the same toad model were scored as a single event [44]. Attacks were categorised according to the type of predator as: (1) Birds; (2) Mammals; (3) Arthropods and (4) Unidentified. Attempts of predation from birds were recognised by beak prints in the shape of a “U” or “V”, as well as long stripes left on the clay (Fig 3A) (e.g. [42,44,64,65]). Mammal predation attempts were identified by characteristic tooth marks (Fig 3B.), and arthropods by little, subtle jaw prints and incisions left on the clay (Fig 3C) [44,66]. Any mark which did not fit in the categories above was classified as unidentified (Fig 3D). Nevertheless, only bird marks were considered for this work, since mammals and arthropods are not visually oriented for predation and might have been attracted to the models despite their shape or colour and visual cues are the basis of our hypothesis [67–69]. Models not recovered after 72h (n = 6) were scored as missing. The effect of inclusion (assuming bird predation) or exclusion (not assuming bird predation) of the missing models was tested.

We used a binomial Generalized Linear Model to determine if attack rate by birds was influenced by the category of the model, the background type and the interaction between model category and background. To test the significance of the variables we used Likelihood Ratio Tests, starting with the general model (with interaction term) and dropping variables sequentially. All statistical analyses were performed using R version 3.3.3. Results were considered significant when p < 0.05.

## Results

Of the 810 models placed on the forest transects, 86 (10.6%) were attacked (being 5.7% by birds) and six (0.7%) were missing—three normal (two over white board and one over the leaf litter) and three green unken (three over the leaf litter). Birds were responsible for 53.5% (46) of the attacks, of which 41.3% (19) were on normal models, 19.6% (9) on green unken models and 39.1% (18) on red unken models (Table 1). Of the 46 attacks, 63% (29) were above the leaf litter and 37% (17) were on the white background.

**Table 1:** Summary of birds predation attempts on clay models of *Melanophryniscus cambaraensis* in relation to body and background.

Toad	Normal		Green unken		Red unken		
**Background**	White	Leaf litter	White	Leaf litter	White	Leaf litter	Total
**Attacked by birds**	7	12	5	4	5	13	46
**Not attacked by birds**	126	122	130	128	130	122	758
**Attack rate**	0.053	0.089	0.037	0.030	0.037	0.096	–

Missing models were not included in the table. Attack rate = attack by birds/total of category.

**Fig 4:**
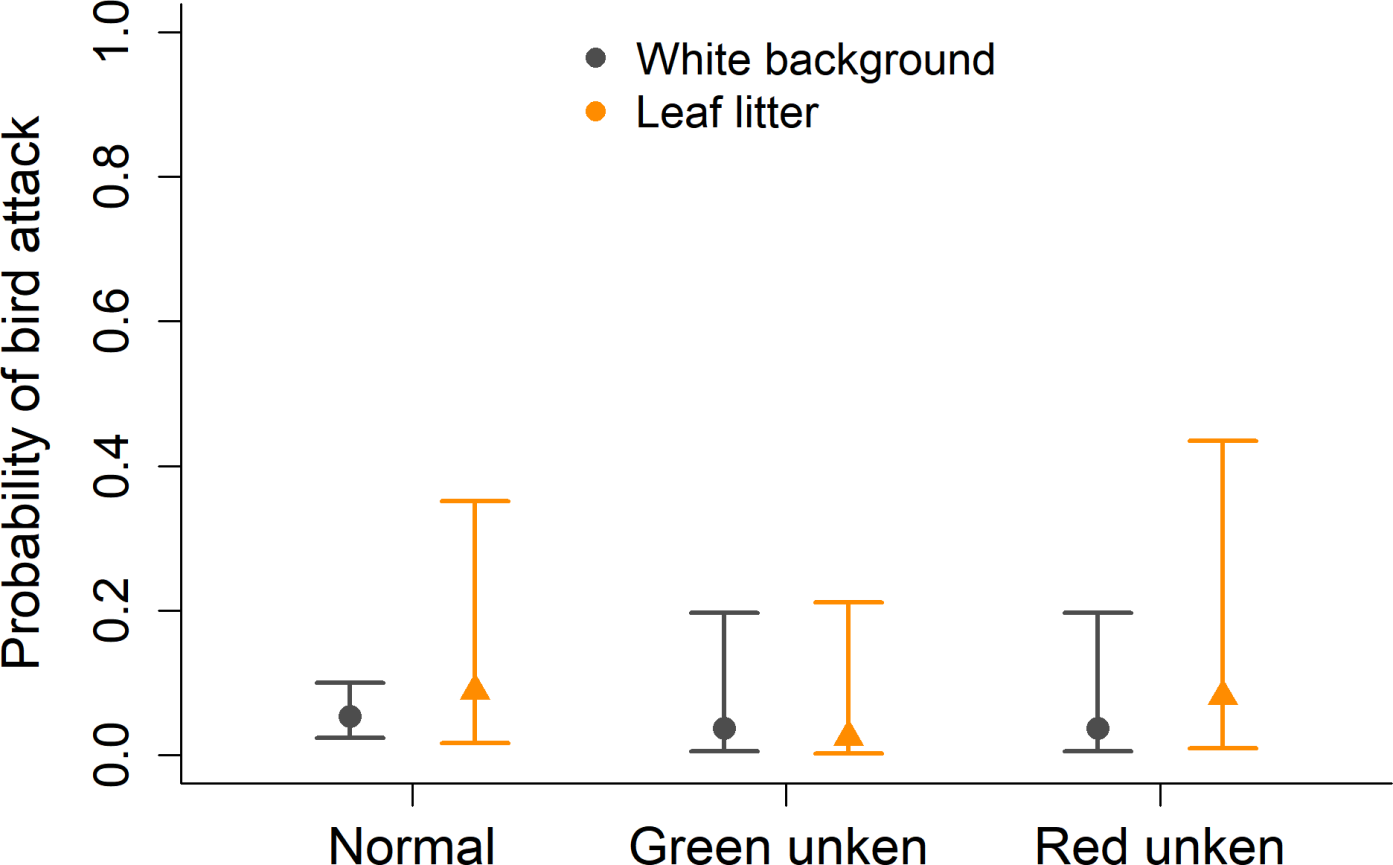
Estimated probability of bird attack on clay models of *Melanophryniscus cambaraensis*. Three toad posture treatments (normal, green unken and red unken) and two background types (white or leaf litter), excluding missing models. Lines represent 95% confidence intervals around the mean.

When missing models were excluded from the analysis, the interaction between model category and background did not have a significant effect on which models were predated by birds (χ^2^ = 2.01; p = 0.37). The toad model categories alone were also not a predictor of bird predation (χ^2^ = 4.59; p = 0.10). When comparing background types, a higher number of clay toads were attacked in the leaf litter, however this difference was not significant (χ^2^ = 3.42; p = 0.06) (Fig 4).

When missing models were included as predation attempts, the interaction of model category and background was not significant (χ^2^ = 1.00; p = 0.61). There was also no significant difference between models (χ^2^ = 3.30; p = 0.19). However, the type of background was a predictor of bird predation (χ^2^ = 4.07; p = 0.04), showing that toads on the leaf litter were on average 1,7 times more likely to be attacked than models placed on the white board.

Mammals, arthropods and unidentified predators were responsible for 7.7%, 6.6% and 32.2% of the total attacks, respectively. Besides that, only five out of 90 models were attacked on the breeding site, two by mammals and three by arthropods.

Even though our experiments were not originally designed to evaluate preferences for body parts by the predator, we consider it relevant to present our results here for the benefit of future experiments. In the models placed in the forest, the normal models received about 70% of the bird attacks on the anterior part of the body, especially on the head and eye region. Another 26% of the attempts were on both anterior and posterior region and 5% only on the posterior region. When comparing this to the position of the attacks on unken models, only 30% of the attempts were on the anterior region, 18% on the posterior and 52% in both.

## Discussion

Our results suggest that attacks by birds on the three different models occur at the same rate. Therefore, these results do not support the working hypotheses that unken reflex behaviour and red colouration in *Melanophryniscus cambaraensis* are efficient warning signals against predation attempts by birds. However, we found evidence that the static unken position reduces the ability of the predator to identify the toad’s head and therefore may reduce the consequences of predation attempts.

Several studies with clay models have suggested that organisms with aposematic colouration have lower predation rates than the non-aposematic organisms (e.g. [14,44,64,66,69]). However, recent studies using Dendrobatidae models also found, similar to our results, that aposematic colouration was not a predictor for the rate of attacks from visually oriented predators [42,70]. A lack of difference in the number of avian attacks on the cryptic and the local aposematic forms of *Dendrobates tinctorius* was also reported, however there was a difference in the attack rate when an unknown aposematic models were introduced [61].

Although the model type was not a predictor of bird attack rates on *Melanoprhyniscus cambaraensis*, the rate of attacks on normal and red unken toads was twice than on green unken toads. These results may be explained by the absence of the green unken phenotype in the environment, inducing a neophobic reaction in the predators [70]. Alternatively, it may be explained as a result of differences in detectability. The red warning colour in the red unken models and the eyes in the normal model might have increased detectability by the predators [71,72]. Recent studies reinforced that birds can be very effective at discriminating similarly shaped objects as they can distinguish a toad from a fruit of similar size, colour and shape [69]. Therefore, we consider that the attacks on the red unken models were the result of a choice and not a mistake.

The effectiveness of aposematic signals depends on the predator`s learning ability and the intensity of the association between the signal and the perceived unprofitability of the prey [8–10]. *Melanophryniscus* spp. contains lipophilic alkaloids sequestered from its diet [73–75] and biosynthesized bufadienolide-like compounds and indolealkylamines as a chemical defence [48]. Therefore, the warning mechanisms (unken reflex and colouration) can be assumed as honest signals of unprofitability [76,77]. However, the effectiveness of the aposematic signal is expected to be higher in prey with high population densities, which are frequently encountered by predators [6,9,78]. The population density of *Melanophryniscus cambaraensis* in the study area is usually low and they are mainly found in the forest, except during reproductive events when the species can be found in aggregations at the breeding site at the edge of the forest. Despite this, the species is spatially very rare, since only one small population is known for the area [56]. This rarity can result in a weak warning signal due to reduced learning opportunities for predators. The lack of other aposematic anurans in the region ([56], Garcia, unpublished data) may also weaken the strength of the signal. We suggest that further studies should investigate the effect of aggregation on bird attack rates. If our assumptions about the strength of the warning signals are correct, we expect to find consistent lower rates of attack in the breeding sites.

When excluding the missing toads from our analysis, the background was found not to be a significant predictor of bird attacks. On the other hand, when including the six missing toads, the rate of attacks is higher on the leaf litter and does not change on the white background. Studies have reported the same pattern for other amphibians [42,64], snakes [44] and moth caterpillars [79], finding a lower bird predation rate when exposing models to high-contrast backgrounds. These results may be explained by a neophobic reaction of predators to white backgrounds [70] and therefore demonstrate that the choice of background is relevant and must be considered for the design of further studies.

One remarkable result of our study was the difference in the rates of bird attacks on body regions. Normal models were attacked 2.3 times more on the head than the unken models. Previous studies have also reported higher attempts of predation on the head region when it could be identified by different morphology or by the presence of eyes, but not in models that lacked this differentiation [71,72]. The lack of conspicuous difference between the anterior and posterior regions in the unken models used in this study may have confused the predators, which were not able to identify the head region. Hence, we suggest that the unken reflex position reduces the ability of the predator to identify the head region of the toads, which may reduce the chances of death or serious damage to the eyes, especially because birds probably perceive the unpalatability of the animal after the first bite. In this sense, the static unken position could function as a deflection mechanism [2], that might allow the prey to avoid mortal injury by directing the predators’ attacks towards a less-vital body area.

In *Melanophryniscus cambaraensis* the presence of conspicuous colouration together with a static unken reflex did not reduce the number of attacks by visually oriented predators. However, a study with static and moving Dendrobatidade models indicated that movement could be important for predicting a predator’s decision to attack or not [66]. We suggest that the strongest anti-predator mechanism in this species may be a synergistic association between four distinct mechanisms: colouration (aposematism), unken reflex movement (deimatism), unken static position (deflection) and secretion of skin toxins (chemical defence). If this is true, the unken reflex should be further investigated concerning its possible deimatic effect which may be independent of the other anti-predator mechanisms and necessary to render the anti-predatory signal effective.

## Supporting information

S1 Table Bird attacks on clay models. **Table containing original data used for the analysis of the bird attacks on the clay models placed in the forest.** In the Bird Attack column: 0 = not attacked and 1 = attacked. In Missing Model column: 0 = not missing and 1 = missing. Model column: number of models; Background column: type of background where the model was placed (white or leaf litter); Transect column: number of the transect where the model was placed; Block column: number of the block where the model was placed; Category column: model type category (normal, red unken or green unken).
(XLSX)
